# "The Hospital" Nursing Mirror

**Published:** 1900-01-13

**Authors:** 


### "The Hospital" Nursing Mirror.

Being the Nursing Section of "The Hospital."

[Contributions for this Section of "The Hospital" should be addressed to the Editor, The Hospital, 28 & 29, Southampton Street, Strand, London, W.C., and should have the word "Nursing" plainly written in left-hand top corner of the envelope.]

### Notes on News from the Nursing World.

### THE NEW ROYAL RED CROSS NURSE.

The Queen has conferred the decoration of the Royal Red Cross upon Miss E. Ryan, superintendent of the Army Nursing Service, in recognition of services rendered in Malta in connection with the nursing of the sick and wounded from Crete. The coveted distinction has been thoroughly well earned by Miss Ryan, whose devotion to her work is well known. She is still engaged in nursing at the Military Hospital, Valetta, about which we hope to publish an article next week.

### NURSING CONVALESCENT SOLDIERS IN CONVALESCENT HOMES.

It is obviously a point of great importance and interest whether the nurses who will be employed in the convalescent homes which are being prepared in various parts of the country for our convalescent soldiers will necessarily have to belong to the Army Nursing Reserve. From inquiries made at the War Office and the Army Medical Department, we learn that the authorities have decided that, to use the words of the Under Secretary of State for War, "it will not be required that nurses in private convalescent homes should belong to the Army Nursing Reserve. It is not considered that men who are fit to go to such homes will require much nursing, and it is not probable that the War Office inspection will be directed to the nursing staff." This is sufficiently definite, and taken in conjunction with the statement made in Victoria Street, that "the War Office will not furnish nurses for the convalescents in private homes, but will be willing to allow the soldiers to avail themselves of voluntary assistance," justifies the conclusion that there may be an opening for nurses who are able and willing to give their services gratuitously, but does not warrant the assumption that any payments will be made by the Government to nurses outside the Army Nursing Reserve. Information about the convalescent homes, and the arrangements for nursing, should, however, be sought from the Soldiers and Sailors' Help Society, 17, King Street, St. James's, S.W.

### SCARCITY OF NURSES AT WYNBERG HOSPITAL.

Writing to *Guy's Hospital Gazette*, Mr. Fremantle, who is with the Army Medical Corps in South Africa, makes the following comments on the nursing :—

"As to the nursing, I can only say that the whole system here appears to be entirely inadequate, and that for no reason, since there are a thousand civilian nurses in England and the Cape willing and thirsting for the work ; and there are few things for which the taxpayer would so willingly pay. Hitherto there have been seven sisters on day duty, and two on at nights for the whole of our 600 beds, spread out over a space at least a quarter of a mile in diameter each way, and on the side of a hill. Practically the whole nursing is meant to be done by orderlies, two to each hut of 25 beds; and these orderlies have to go round with the officer each day ; help in the dressings; go and order the meals and fetch them from the cook house ; scrub the ward and the furniture and appliances ; fetch the medicines from the dispensary ; and, in addition, do the nursing. This being the case I went into one of my huts on Sunday and found that my head nurse, so to speak, was confined to cells for being drunk on the previous night ; and the one remaining orderly said that was nothing, as he had had eleven days not long ago, and lost his corporal's stripes after fourteen years' service ! And then the day orderlies have to take it in turn to be on night duty as well as day ; there are at present so few orderlies in the place that they practically have to be on night duty as well as day every other night. What can one expect under such conditions? Little wonder if we get bed-sores. Of course, with a good orderly, and patients able and willing to help, as in one of my huts, all goes fairly well ; but even so the staff is quite inadequate. We ought to have one nursing sister and four orderlies on for each hut, and then all would be well, even allowing for an occasional night in the cells, which, I suppose, is a necessity."

We call the attention of the War Office to the statements of Mr. Fremantle, which materially confirm the conviction that the appointment at the front of a great many more nurses, not necessarily of the Army Service or the Army Nursing Reserve, is urgently demanded.

### THE DIFFICULTIES OF NURSING ON A HOSPITAL SHIP.

Our correspondent on board the "Spartan" writes from Capetown as follows : "During our three weeks in Durban Harbour our invalids got on wonderfully well. Heaps of luxuries and flowers were brought on board by visitors. We had a good passage to Capetown, but after being in dock so long we were all very seasick on first getting out to sea again, and had to make occasionally rapid exits from the wards and lie down between visits. Nursing in a rolling or pitching ship, with every object seeming alive and lively, is somewhat different from nursing on *terra firma*—the experience has to be gone through to be realised. We arrived outside Capetown at half-past seven a.m., and did dressings and gave patients' their breakfast early expecting a speedy landing, but we could not get into the docks till twenty-five minutes past four p.m. In 50 minutes we put out again, but even in that short time numerous visitors came on board."

### PRINCESS SALM SALM.

In reference to our statement on December 30th that the Princess Salm Salm had undertaken the superintendence of the nursing on the Boer side, Mr. Charles Heneage, of Felstow Hall, Louth, writing to us under date of January 5th, says : "My permission to my wife, H.S.H. Princess Salm Salm, to go out to Pretoria in charge of the sick and wounded combatants *and prisoners* is withdrawn, owing to the incapacity of the War Office, *with a civilian presiding over our armies*, to meet my views, who have had experience in *three* wars." The italics are not ours.

### A CONTRIBUTION FROM NATAL TO THE JUNIUS S. MORGAN BENEVOLENT FUND.

It is pleasant to learn that the Junius S. Morgan Fund has received the sum of £3 4s., collected by Nurse Mary Roberson, of Durban. This amount, together with previous remittances, makes up £5 15s. 6d. which Miss Roberson has either collected or given on behalf of her poorer sisters. The fact is all the more remarkable bearing in mind the present state of affairs in Natal. In connection with this announcement of

Nurse Mary Roberson's kind efforts on behalf of the Benevolent Fund, we are asked by the Hon. Secretary of the Fund to remind members of the Pension Fund that all did not send their contribution of 1s. during the past year, as it was hoped they would do, and consequently the contributions from the nurses to their Benevolent Fund will show a falling off in the coming report. Contributions are now due for the present year.

### THE RESIGNATION OF MISS NOTT BOWER.

We greatly regret to announce the resignation by Miss Nott Bower of the post of matron of Guy's Hospital, in consequence of ill-health. Miss Nott Bower had not been well for some time past, but at the last she broke down rather suddenly, and in consequence of peremptory medical orders, her departure became inevitable. Commencing her training at Guy's in 188[illegible], she filled the position of Sister in Dorcas Ward for five years, when she was appointed matron of Huddersfield Infirmary. But in 1889 she returned to Guy's in order to take charge of the Private Nursing Institution, becoming matron of the hospital on January 25th, 1893. Under her auspices, the present excellent system of longer hours off duty, the new club house at Honor Oak, the present system of sisters' appointments, and many other improvements in a nurse's life were effected. On her departure she was presented by the treasurer with a gold watch and chain as a mark of esteem in which she was held at the hospital; and at a meeting of the governors a resolution of regret at her unavoidable resignation was passed unanimously. Miss Nott Bower has always been a warm supporter of the Royal National Pension Fund, and no allusion to her distinguished career would be complete without mention of her valuable services on the committee of the Junius S. Morgan Benevolent Fund. She was the most regular attendant at the meetings, and she will be much missed by her former colleagues. We trust, however, that after the prolonged rest which has been prescribed for her Miss Nott Bower may be able to undertake some less arduous work than that which, to the sorrow of all who know her, she has been reluctantly compelled to relinquish.

### THE NEW MATRON OF GUY'S HOSPITAL.

The new matron of Guy's Hospital is Miss Esther Harriet Young, who has been assistant matron since January, 1897. She was trained at Addenbrooke's Hospital, Cambridge, from October, 1886, to November, 1887; and at the London Hospital from 1887 to 1888. From 1889 to 1890 she was nurse at Gordon House Home; from 1891 to 1892, private nurse at the Home for Nurses, Fitzwilliam Street, Cambridge; and from 1892 to April, 1896, assistant matron at Addenbrooke's Hospital. For the remainder of 1896 she was a monthly nurse at the General Lying-in Hospital. Sister Esther is very popular at Guy's, and the fact that she has carried off one of the great prizes of the nursing profession has been hailed with the utmost satisfaction in the hospital, as we are convinced it will be throughout the whole of the nursing world.

### ST GEORGE'S INFIRMARY, FULHAM ROAD—CONFIRMATION OF OUR CORRESPONDENT'S STATEMENTS.

Elsewhere we print letters from "An Indignant Probationer," and from other probationer of St. George's Infirmary, Fulham Road, which undesignedly confirm in a remarkable manner several of the statements made by our correspondent last week. "Our grievance," says "An Indignant Probationer," "is overwork," but "it does not concern the public." It will be remembered that our correspondent commenced by calling attention to this particular point. Now we have the assurance of two of the nurses themselves that the staff is overworked; and the grievance does concern the public. For St. George's Infirmary is a public institution, which could not exist a day without the support of the public rates. It will be observed that in respect to the other charges made by our correspondent, the protesting probationers merely express an opinion. They do not challenge the figures given in respect to the patients' food; they do not say at what hour the last meal of the nurses in the day takes place. Neither do they dispose of the statement about the nurses being obliged to wear damp clothes, although one affirms that the occurrence was exceptional. There is no denial in their letters of the urgent need of a communicating bell connecting the upper and lower wards; and no attempt to prove that the arrangements of the wards are not deplorable. We print their letters with pleasure, although they bear a strange family resemblance, and we do not demur to the contention that the matron and the doctor take the greatest interest in the staff, and endeavour to maintain the highest standard of nursing possible for their patients' comfort, but we must remind them that no sort of reflection was made by our correspondent upon either of these officials. Perhaps it has not occurred to "Major" that there must be something wrong in an institution which possesses, as she avers, "a family skeleton;" and her assertion that "the officials cannot be expected to reform the establishment in as short a time as it takes to form outside opinion," is, of course, a confession that the management needs reform. The naïve conclusion of "A Third Year Probationer's" letter that "we have appealed to the committee but not to the public," speaks for itself. But, after all, it is from the parish authorities of St. George's, Hanover Square, not the probationers in the Infirmary, that the defence, if defence there be, should come; and they are utterly mistaken if they imagine that the pleas of a fraction of the 60 nurses employed against publicity being given to the statement which they assist to corroborate will absolve those who are really responsible from blame.

### THE NURSES OF THE GLASGOW ROYAL INFIRMARY.

At the annual meeting of the managers with the nurses of the Glasgow Royal Infirmary last week, Mrs. Strong, the matron, and her staff were the recipients of many well-merited compliments. The Lord Provost having observed that "nurses required warm hearts, cool heads, and clever hands," said that "there was ample testimony that the nurses of the Glasgow Infirmary were equipped with all these requisites for the efficient discharge of their duties." Mr. Hugh Brown dwelt on the manner in which "Mrs. Strong and the nurses did their best to overcome difficulties," and alluded to the excellent training given; while Sir William Gairdner commented, in eloquent language, on the spirit which he believed largely animated nurses of the present day. He maintained that the great reform in nursing during the last 40 years had its origin in a desire to benefit mankind, and continued :—

"That noble woman—Miss Nightingale—acted upon this principle. She had a good social position, but she was willing to expose herself to danger in order to redeem the nursing of our soldiers and sailors from the depths into which it had fallen. From that moment there had been steady improvement. Of no institution could that be said more truly than the Royal Infirmary. It had in those early days a great staff of nurses who were good, bad, and indifferent. Some of them were as good as could possibly be conceived, and in Edinburgh much earlier he could recall fine old women full of experience and kindly feeling; indeed, they were amongst the best nurses he had known; but they were surrounded by a terribly poor lot. What a great advance had been made, brought about by inspiring young women who did not need to go out at all with the spirit of our Lord. It was by this that nursing was redeemed from the state described by Dickens."

### A NURSING ASSOCIATION FOR WESTMORELAND.

It is proposed to form a nursing association in Westmoreland, and at an influential meeting the other day, Lady Lonsdale in the chair, Miss Ellis, superintending secretary for the Cumberland Nursing Association, gave an interesting account of the work which is going on in the neighbouring county. Miss Ellis stated that the nurses in Cumberland went about on bicycles, but pointed out that those machines could not be used in ali weathers, and therefore maintained that in no case should a nurse have to travel more than five miles from her place of abode. The plan she advocated was to fix on a number of small villages, group them together, and place a nurse in the most central. She strongly advised the Westmoreland people to go in for "a fully-trained nurse." Admitting that doctors are very kind in showing less qualified nurses what to do, she urged that a fully-qualified nurse was best, "because there was something in her training which helped patients, and there was no doubt that persons recovered more quickly under the care of a trained nurse than without such looking after." Dr. Haswell endorsed Nurse Ellis's counsel, and observed that doctors were always pleased to work with the district nurses. The movement has been so well received in Westmoreland that it does not seem premature to assume that an influential association will soon be in existence.

### "NURSES" AT £15 A YEAR.

Mr. Preston Thomas, the Local Government Board inspector, has been giving the Guardians of the Bridgwater Union a nice little wigging. For some time there was only one nurse for fifty patients, and when at length the Guardians under pressure appointed two more, Mr. Thomas reminded them, that they offered as salary "the wages of a scullery-maid." There were cries of "No!" but Mr. Thomas, of course, insisted that for a salary of £15 a year it is obviously impossible to get anyone with skilled knowledge. The reverend vice-chairman, who seems to be proud of the scullery-maid standard, informed the inspector that the Board had "less difficulty in obtaining assistant nurses at £15 a year than in getting a head nurse at £35 per annum"; but Mr. Thomas effectively rejoined, "You can get people to come here for £15, but not nurses." Mr. Thomas proceeded to refer to the extraordinary number of changes which had been made in two years, and insisted that the fact of three head nurses and five assistant nurses resigning in that period showed there was "something which operated unfavourably." But what can be expected from a Board of Guardians who in these days choose to remain under the delusion that the services of a nurse can be obtained for £15 a year?

### EVENING HYMN FOR HOSPITAL NURSES.

Mr. Henry Froude, of the Oxford University Press, has published an "Evening Hymn for Hospital Nurses," dedicated to Princess Christian. The words are by the Rev. S. Harvey Gem, and the music by Dr. Varley Roberts, of Magdalen College, Oxford. The name of the composer is a guarantee of the excellence of the music. We give the first four verses of the words :—

"Of old before the Temple's shrine
The Priests their watches kept;
While safe beneath the Wing Divine
The Holy City slept.

Still gleam'd the lamps with radiance dim,
Still glow'd the altar's fire,
As through the gloom in midnight hymn
Rose up their high desire.

And is not this Thy Temple, Lord,
Where sick and dying lie?
If only Thou Thy grace afford
Our work to sanctify!

The watchers to the service vow'd
Of those by pain oppress,
May worship, though no knee is bow'd
By guarding them in rest."

### SHORT ITEMS.

The lady superintendent and nursing staff at Netley will shortly have the gratification of seeing the Queen, who has signified her intention of paying a visit to the hospital. — The bequests of the late Mr. Manasseh Gledhill, of Brickfield, Rusholme, Manchester, a director of Armstrong, Whitworth, and Co. (Limited), include £250 to the Manchester and Salford Sick Poor and Private Nursing Institution.—The new vicar of Burton-on-Trent, the Rev. H. B. Freeman—who will be remembered as curate of St. Anne's, Soho—has been preaching an eloquent sermon on behalf of the Burton Nursing Institution, in which he referred to the fact that three night nurses, as well as five day nurses, are supplied. —Next Thursday evening there will be an entertainment for the nurses and their friends at the National Hospital for the Paralysed and Epileptic, Queen Square, Bloomsbury. This is one of the annual course of winter entertainments which have been going on for 30 years.—An entertainment for the out-patient children of the Hospital of St. Francis, New Kent Road, was given on Friday to more than 170 boys and girls. Songs, recitations, and acted nursery rhymes kept the children well amused, the songs with choruses being joined in with so much zest and tune as to convince the interested onlookers of the fallacy of the statement that the English are an unmusical nation.— An entertainment of an excellent character was provided for the amusement of the patients of the Cancer Hospital, Fulham Road, on Thursday evening by Mr. W. Ernest Miles (one of the honorary surgeons of the hospital) and his friends. The recitation of the "Absent-minded Beggar," by permission of the *Daily Mail*, was given by Mr. H. Cecil Kenway, and was received with much applause, especially as it was announced that he was about to leave England for the seat of war.—A working party in aid of the "Absent-minded Beggar" Fund, meets every Wednesday at the Trained Nurses' Club, 12, Buckingham Street, Strand, between two and four o'clock. Nurses who can spare a little of their time to help in this way will be welcomed.

### Lectures to Nurses.

By CARSTAIRS DOUGLAS, M.D., B.Sc.Edin., Professor of Medical Jurisprudence, Anderson's College Medical School, and Dispensary Physician, Samaritan Hospital, Glasgow.

### DIETETICS AND PHYSIOLOGY OF DIGESTION.

ONE of the most important of all the duties that fall to the hands of a nurse is the administration of nourishment to her patient. The proper feeding of the sick embraces a variety of important points, such as the nature of the food and its suitability for the illness, the times and methods of administration, the modes of preparation, and so on, and before a nurse can lay satisfactory claim to competency in this department, she must have a clear, though not necessarily profound, knowledge of the processes of digestion and of the needs of the organism, in health and disease, in respect to various kinds of food-stuffs.

Let us therefore, in the first place, devote our attention to a general survey of the anatomy and physiology of the entire digestive tract; then to a consideration of the various groups of foods and the mode of digestion of these in detail; and, lastly, to the blending together of food materials in suitable combinations and their administration to the patient, *i.e.*, dietetics proper.

### I.—GENERAL ACCOUNT OF THE ANATOMY AND PHYSIOLOGY OF THE DIGESTIVE TRACT.

Food is first of all received into the mouth, where, in the case of solid or semi-solid masses, it is reduced to a pulp by the cutting action of the incisor teeth, the tearing action of the canines and bicuspids, and the grinding of the molars. This process is rendered easier by the admixture of saliva and mucous secretion with the food bolus, while the action of the tongue and cheeks keeps the ingested morsel in constant motion. This reduction of food to a pulp is an important preliminary to digestive action, and this explains why persons with bad or deficient teeth so often suffer from dyspepsia.

In addition to this mechanical treatment of the food, certain very important chemical changes take place in the mouth under the influence of the saliva. This fluid is secreted by three pairs of salivary glands—(1) the parotids lying upon the cheeks, below and in front of the ears; (2) the sub-maxillary placed below the lower jaw; and (3) the sub-lingual situated underneath the tongue. The ducts from these six glands open within the mouth, and the secretion becomes especially free when a morsel of food has been taken.

Saliva itself may be conveniently studied by allowing a little to flow into a conical medicine glass, when it will be seen to be a slightly opalescent mucoid fluid mixed with many air-bells. After it has stood for some time it will appear more opaque from the gradual precipitation of calcium carbonate, or common chalk. On the reaction being tested it will be found to be alkaline, while a little of the fluid examined under the microscope shows many air-globules of varying size, flat epithelial cells from the roof of the mouth, and round cells termed salivary corpuscles. It is not necessary that we should study the composition of saliva very minutely; suffice it to say that it contains various organic and inorganic ingredients, and that of these by far the most important is a ferment named *ptyalin*. Now it is necessary that we understand exactly the meaning of the terms *ferment* and *fermentation*. A ferment is a body which, when present in an organic mixture, fluid or solid, is able to induce certain special alterations in it, usually in the direction of decomposition, and often with the evolution of gas. A familiar example of fermentation is where malt- or grape-sugar is broken up under the action of the yeast plant, which acts here as the ferment, into alcohol and carbonic acid gas. Another familiar example is the souring of milk by the lactic acid bacillus. In each of these cases cited the ferment is an organism of definite shape, which can be grown under suitable conditions, can be studied microscopically, and so constitutes what is termed a formed ferment. But there is another class met with, especially in the digestive fluids, which are of no definite shape, act when present even in very small traces, and seem themselves frequently to be unaffected by the process—such are termed unformed ferments, and of these the ptyalin in the saliva is an excellent example. This ferment acts upon the starch in our food, converting it rapidly into a sugar named "maltose" and a body named "dextrin," which is, as it were, half-way between starch and sugar. We shall consider the action of this salivary ferment in greater detail later on, when considering the digestion of various food-stuffs; let us meantime pursue our description of the alimentary canal.

The food mass, after its thorough impregnation with saliva, is handed backwards by the tongue and cheeks till it reaches the pharynx, an expanded, bag-like portion of the digestive canal lying behind the nose and mouth, and reaching as far down as the fifth vertebra of the neck. Here the food is grasped by a set of muscles called the "constrictors," and these hand it on to the gullet, which, in its turn, gently but steadily passes it into the stomach. Liquids, it may be mentioned, travel much faster.

The gullet, or œsophagus, is a muscular tube, ten to twelve inches in length, reaching from the pharynx to the stomach, and lying in the posterior part of the thorax in front of the vertebral column. The muscles of which its walls are composed are involuntary, *i.e.*, beyond the control of the will. We find accordingly, then, when the food mass has entered it, it cannot be brought back into the mouth at the desire of the person swallowing. The gullet is not infrequently the seat of stricture or narrowing, in many cases due to cancer, in others to damage done to its walls by the swallowing of corrosive fluids. Under these circumstances the patient has great difficulty in swallowing, especially in the case of solids, and often one may find that food is regurgitated or thrown back into the mouth some minutes after deglutition.

The œsophagus having passed through the diaphragm somewhat to the left of the middle line, enters the stomach, a portion of the digestive tract of such importance as to merit a somewhat detailed description.

The stomach should not be looked upon as an isolated organ, but simply as a dilated portion of the alimentary canal in which the food is subjected to certain special processes before it enters the intestine. It is of the shape of a pear bent upon itself, so that it presents a large end, termed the cardia, placed to the left side, and a narrow extremity—the pylorus—pointing more or less towards the right; a greater curvature looking downwards, and a lesser directed upwards, as well as anterior and posterior surfaces, whose names explain their position. The emptier the organ is the nearer do the anterior and posterior walls approach to one another. The pyloric extremity is not far (about three inches) from the entrance of the œsophagus, and the greater curvature, in health, should not be nearer than one inch to the umbilicus.

The average length of the stomach from right to left is 10 inches, the depth between the curvatures 4½ inches, while the capacity for an ordinary individual is about 2 pints (40 oz.) It is provided with four coats: (1) The peritoneal covering, (2) the muscular coat, (3) the submucous, and (4) the mucous membrane. Of these the second and fourth are the most important. The mucous coat contains a large number of glands, some of which secrete mucus, while others, with which we are specially concerned, secrete the gastric juice, which is so important in digestion.

### A Question of Immediate Importance to all Trained Nurses.

THE NURSES' WHO'S WHO?

Last week, in reply to the inquiry of a correspondent who wanted, naturally enough, to know why, in our article on "The Princess of Wales's Nurses for the War," we gave a full account of the training of several nurses and omitted to supply any details in respect to the others, we stated that we published all the information we possessed, and we urged that all nurses who wished to enjoy the obvious advantages of having their training and past experience stated when they are appointed to posts of importance, should not fail to send in full details of their career for publication in the next issue of "Burdett's Official Nursing Directory."

But the imperative need of this step in the interest of nurses has been further impressed upon us, since, by a difficulty which, we know, actually arose in ascertaining the training of a lady whose name had been mentioned in connection with nursing the sick and wounded at the seat of the war, and which, it was found on reference, was not included in the official directory. It is always a great disadvantage when the antecedents of a nurse can only be obtained by hunting for them, and very often the chase is either not undertaken, or it is given up in despair.

Suppose, for example, that a doctor is in immediate want of a nurse and hears of one who is highly recommended, but the person recommending is ignorant about her training. If the doctor can take down from his book-shelf "Burdett's Official Nursing Directory" and ascertain that she has had a creditable professional career, he is in a position to telegraph at once for her services. But if there is no record in this "Directory," he has either to lose a lot of time in obtaining the particulars, or he more likely engages someone else whose name and career do appear there. Or a case of sickness suddenly arises in the home, and it is realised that the doctor and the nurse must be sent for simultaneously. There is "Burdett's Official Nursing Directory" for consultation, and from among the names of nurses engaged in private nursing it is not difficult to make a satisfactory selection. At any rate, there is the certainty that, whoever is chosen, no error will be made in regard to her qualifications.

Just now, moreover, when at any moment more nurses may be wanted at the shortest notice for the front for nursing the wounded on their way home, or for nursing the convalescent when they have arrived, the drawback of not being entered in "Burdett's Official Nursing Directory" is particularly serious. It is inevitable at such a crisis as the present that recommendations given should often be unaccompanied by details, and yet the speediest possible choice is indispensable. Therefore, unless the career of the nurse can be learnt forthwith she runs a great risk of being left out in the cold, no matter how excellent her training or how admirable her qualifications may be for the work. We receive every week a number of letters from nurses who desire to go out to South Africa or to work on board the hospital ships. But many of them have, so far, entirely failed to grasp the importance of entering themselves in "Burdett's Official Nursing Directory."

There are those who advocate the State registration of nurses as the only means of protecting the nursing profession from black sheep—half-trained persons, and absolute ignoramuses. But "Burdett's Official Nursing Directory" is designed to afford this protection, and would afford it entirely if every properly-trained nurse would make a point of applying for admission to its pages. Every hospital or private nurse who has received thorough training is freely supplied with a form of application, and no charge whatever is made for insertion of the particulars communicated.

"Burdett's Official Nursing Directory" is a directory exclusively compiled from information officially certified, and its compilation is under the direction of a committee of doctors and hospital matrons. There is the nurse's career in print; it speaks for itself; it can be investigated; the statements, if challenged, can be proved or disproved. We are so convinced of the disabilities under which nurses who are not entered in "Burdett's Directory" labour, especially in time of war, or in the always possible outbreak of an epidemic, that we append in full the form of application which nurses who wish their names to appear in the subsequent editions should send at once to the Acting Editor, "Burdett's Official Nursing Directory," 28 and 29, Southampton Street, Strand, London, W.C. :—

To be filled up by the Nurse to whom the particulars relate.

(1) Name in full, and Address—

(2) Present Occupation— Date of entering upon it—

(3) Particulars of Nursing Career in the following form :—

| | | | | |
|---|---|---|---|---|
| Probationer, at | Hospital from | 18 | , to | 19 |
| Staff Nurse, at | Hospital from | 18 | , to | 19 |
| at | from | 18 | , to | 19 |
| at | from | 18 | , to | 19 |
| at | from | 18 | , to | 19 |

(4) Certificates stating in each case at what Hospital or Institution training was received, and giving the dates of the commencement and end of training :—
General Training Certificate received at Hospital for years' training.
Midwifery Certificate—
L.O.S. Certificate—
Monthly Nursing Certificate—
Massage Certificate—
Medico-Psychological Certificate—

(5) List of Medals and Badges held, if any—

(6) Any qualifications or experience beyond what is given above?

### Appointments.

Leeds Union Infirmary.—Miss Joanna Inglis has been appointed Matron. She was trained at the National Hospital, Queen Square, W.C., and the London Hospital, and has since been night superintendent at the General Hospital, Wolverhampton, and assistant matron at Poplar and Stepney Sick Asylum.

Dawlish Infirmary.—Miss A. K. Gough has been appointed Matron. She was trained at the Bristol Royal Infirmary, and has been matron of the East Molesey and Hampton Court Cottage Hospital, matron of the Queen Victoria Nursing Home, Freshford, and superintendent nurse of the district. Miss Gough holds the L.O.S. certificate.

Boscombe Hospital, Bournemouth. — Miss Florence Worley has been appointed Matron. She was trained at St. Bartholomew's Hospital, and has since been sister at Swanley Hospital Convalescent Home, and during the past three years sister at the "Dreadnought" Seamen's Hospital, Greenwich.

Blaby and Wigston Magna Infectious Hospital, Leicester.—Miss Margaret Beesley has been appointed Matron. She was trained at the Parish Infirmary, Liverpool, and has since been head nurse and deputy matron at the City Hospital, North Liverpool.

Royal Victoria Nursing Home, Ascot.—Miss Ida C. Mackintosh has been appointed Matron. She was trained at Sunderland Infirmary, and has since worked as district nurse for the North London Nursing Association in Holloway.

Guy's Hospital.—Miss Elizabeth Hodges, "Sister Mary," has been appointed Assistant Matron. She was trained at Guy's Hospital, became staff nurse in May 1894, out-patient and holiday sister in 1896, and sister in January, 1898.

### The Princess of Wales's War Fund.

We publish to-day a further list of subscriptions from nurses to the Princess of Wales's War Fund, and remind our readers that the subscription is limited to five shillings. All subscriptions should be addressed to the Manager, Office of The Hospital, 28 and 29, Southampton Street, Strand, London, W.C., and the name and address should always be given. The list will close at the end of the month.

Amount already acknowledged, £155 16s. 3d.

| | s. | d. |
|---|---|---|
| A. K. Frier ... ... | 5 | 0 |
| Nurse Thomson ... | 2 | 6 |
| H. S. Gibbons, P.F. ... | 5 | 0 |
| Elizabeth S. Attree ... | 5 | 0 |
| L. Hennet, P.F. ... | 5 | 0 |
| E. C McGill ... ... | 5 | 0 |
| Nurse Godfree... ... | 5 | 0 |
| Kate Muller ... ... | 5 | 0 |
| A Winter, P.F. ... | 5 | 0 |
| Edith M. Preece, P.F. | 5 | 0 |
| Eliza Cook, P.F. ... | 5 | 0 |
| Nurse Packe ... ... | 1 | 0 |
| Nurse Woodward ... | 1 | 0 |
| Annie E. Smiter, P.F. | 1 | 0 |
| Margaret Schwappe ... | 1 | 0 |
| Nurse Cook, P.F. ... | 1 | 0 |
| Nurse Lock, P F. ... | 1 | 0 |
| F Goodrich, P.F. ... | 1 | 0 |
| Sister (Helen) Swain, P.F. ... ... ... | 2 | 0 |
| Miss Herbert, P.F. ... | 2 | 0 |
| Sister Thompson, P.F. | 2 | 0 |
| Sister Robinson, P.P. | 1 | 0 |
| Sister Wright, P.F. ... | 1 | 0 |
| Sister Ogle ... ... | 1 | 0 |
| Sister Beer, P.F. ... | 1 | 0 |
| Nurse Haslam, P.F. ... | 2 | 0 |
| R. E. Apted, P.F. ... | 5 | 0 |
| Policy 3,315, P.F. ... | 2 | 0 |
| E. K. Hewlett, P.F... | 2 | 0 |
| [illegible]. J. Lilley, P.F. .. | 1 | 6 |
| Nurse Tucker, P.F. ... | 1 | 0 |
| R. M. Selfe, P.F. ... | 1 | 0 |
| A. A. Headford, P.F... | 1 | 0 |
| Nurse Crisford, P.F.... | 1 | 0 |
| M. Wood, P.F. ... | 1 | 0 |
| T. Taylor ... ... | 5 | 0 |
| G. Sainty, P.F. ... | 5 | 0 |
| Nurse Flint, P.F. ... | 5 | 0 |
| Nurse Jarman, P F. ... | 5 | 0 |
| Nurse Hinton, P.F. ... | 5 | 0 |
| Nurse M. Payn, P.F. | 5 | 0 |
| Essie Gelston, P.F. ... | 5 | 0 |
| Blanche Holman ... | 5 | 0 |
| Nurse Tomlin, P.F. ... | 5 | 0 |
| E. M. A. Guerring, P.F. ... ... ... | 5 | 0 |
| L. Y. Wheatley, P.F. | 5 | 0 |
| Julia Connell ... ... | 5 | 0 |
| L C. Fisher, P.F. ... | 5 | 0 |
| Veronica Richardson... | 4 | 0 |
| Mrgrt. Ringwood, P.F. | 4 | 0 |
| Janet Young, P.F. ... | 3 | 0 |
| Ada Morgan, P.F. ... | 3 | 0 |
| Nurse Maclaren ... | 3 | 0 |
| A. L. Phillips, P.F. ... | 2 | 6 |
| Policy 6,383, P.F. ... | 2 | 6 |
| Cave BrowneCave, P.F. | 2 | 6 |
| Policy 6,130, P.F. ... | 2 | 6 |
| A. C., Policy 4,500, P.F. | 2 | 6 |
| Moira E. Smith, P.F. | 2 | 6 |
| A. C. Clarke, P.F. ... | 2 | 6 |
| [illegible]. L. Hedderley, P.F. | 2 | 6 |
| E. Thompson, P.F. ... | 2 | 6 |
| Policy 3,692, P.F. ... | 2 | 0 |
| M. Powell, P.F. ... | 2 | 0 |
| Nurse Celia, P.F. ... | 2 | 0 |
| Nurse Cripwell, P.F. | 2 | 0 |

| | s. | d. |
|---|---|---|
| Elizabeth Peach ... | 2 | 0 |
| Nurse Wise, P.F. ... | 2 | 0 |
| F. I. Claringbold, P.F. | 2 | 0 |
| Jessie H. Clarson, P.F. | 2 | 0 |
| Policy 5,810, P.F. ... | 2 | 0 |
| Sister Sara, P.F. ... | 2 | 0 |
| M. Stone, P.F. ... | 1 | 6 |
| E. F. Ward, P.F. ... | 1 | 0 |
| Nurse Harris ... ... | 1 | 0 |
| Nurse Parker ... ... | 1 | 0 |
| Mabel D. Taylor, P.F. | 3 | 0 |
| S. A. Booth, P.F. ... | 3 | 0 |
| John F. Rubbins, P.F. | 2 | 6 |
| Alice Farnborough ... | 2 | 6 |
| Mrs Cresswell, P.F.... | 2 | 6 |
| A. P. James, P.F. ... | 2 | 6 |
| Sister Margaret, P.F... | 2 | 6 |
| G. Beach, P F. ... | 2 | 6 |
| M. Puxley, P.F. ... | 2 | 6 |
| A. M. Pease, P.F. ... | 2 | 6 |
| Agnes Lyle, P.F. ... | 2 | 6 |
| Mrs. Belton ... ... | 2 | 0 |
| M. Ingham, P.F. ... | 2 | 0 |
| E. Brooks, P.F. ... | 2 | 0 |
| Jane Anstey, P.F. ... | 2 | 0 |
| J. E. Hughes ... ... | 2 | 0 |
| Julia Staples, P.F. ... | 2 | 0 |
| Policy 7,345, P.F. ... | 2 | 0 |
| A. B. Young, P.F. ... | 2 | 0 |
| E. H. Rymer, P.F. ... | 2 | 0 |
| Caroline Swain, P.F... | 1 | 0 |
| Policy 81, P.F. ... | 1 | 0 |
| Policy 1,046, P.F. ... | 1 | 0 |
| E. J. D. and A. C., P.F. | 1 | 0 |
| L H. Wilson, P.F. ... | 1 | 0 |
| Helen Largue, P.F. ... | 5 | 0 |
| Isabel J. Baylor, P.F. | 5 | 0 |
| A. L. Willcos, P.F. ... | 5 | 0 |
| Nurse Ayrton, P.F. ... | 5 | 0 |
| Policy 7,299 (Annie Speck), P.F. ... | 5 | 0 |
| Kate Hale, P.F. ... | 5 | 0 |
| Nurse Chadwick, P.F. | 5 | 0 |
| Miss Alice Tourtel ... | 5 | 0 |
| Nurse Prior, P.F. ... | 3 | 0 |
| Frances K. Gardner, P.F. ... ... ... | 3 | 0 |
| Nurse Flitcroft, P.F ... | 3 | 0 |
| Emily Berkeley ... | 2 | 6 |
| N. Whitfield, P.F. ... | 2 | 6 |
| Kate Nichol, P.F. ... | 2 | 6 |
| Nurse Holker, P.F. ... | 2 | 6 |
| H. Sharland, P.F. ... | 2 | 6 |
| A. R. Webster, P.F. ... | 2 | 6 |
| Wyeleen A. Darrah, P.F. ... ... ... | 2 | 6 |
| Nurse Alexander ... | 2 | 0 |
| K. Wilson, P.F. ... | 2 | 0 |
| G. Redih, P.F. ... | 2 | 0 |
| Maud M. Payne, P.F. | 2 | 0 |
| Ellen C. Hicks, P.F.... | 2 | 0 |
| Nurse Waterman, P.F. | 1 | 6 |
| Nurse de la Chanmette, P.F. ... ... ... | 1 | 0 |
| Policy 3,667, P.F. ... | 1 | 0 |
| Nurse E. Paull, P.F.... | 1 | 0 |
| A. A. Easton ... ... | 1 | 0 |
| Grace M. Plant, P.F.... | 1 | 0 |

| | s. | d. |
|---|---|---|
| Harriet Lovell, P.F. ... | 5 | 0 |
| Janet Butler, P.F. ... | 5 | 0 |
| Laura Gillingham ... | 5 | 0 |
| Policy 5,007, P.F. ... | 5 | 0 |
| A. Bates, P.F.... ... | 5 | 0 |
| M. E. Donaston, P.F. | 5 | 0 |
| Annie Jones, P.F. ... | 5 | 0 |
| M. F. ... ... ... | 5 | 0 |
| A Policy Holder, P.F. | 3 | 0 |
| A. Preece, P.F. ... | 3 | 0 |
| H. M. Good, P.F. ... | 3 | 0 |
| M. Reeve, P F. ... | 2 | 6 |
| H. Warel, P.F. ... | 2 | 6 |
| Nurse Hughes, P.F. ... | 2 | 6 |
| A. M. Darrah, P.F. ... | 2 | 6 |
| M. Revill ... ... | 2 | 6 |
| E. Ruth Clarke, P.F. | 2 | 6 |
| Nurse Pearse ... ... | 2 | 6 |
| M. Cornell, P.F. ... | 2 | 6 |
| Emily Basan, P.F. ... | 2 | 6 |
| Margaret Griffith, P.F. | 2 | 0 |
| Nurse Stanley ... ... | 2 | 0 |
| L. Goodacre, P.F. ... | 2 | 0 |
| E. Jones, P.F.... ... | 2 | 0 |
| Annie Barker, P.F. ... | 2 | 0 |
| Miss Forbes, P.F. ... | 2 | 0 |
| Nurse Adams ... ... | 2 | 0 |
| S. Lambert, P.F. ... | 2 | 0 |
| Fanny J. Daniel, P.F. | 1 | 0 |
| M. A. Bateman, P.F.... | 1 | 0 |
| E. M. Washtell, P.F... | 1 | 0 |
| Charlotte Simmons, P.F. | 1 | 0 |
| Elizabeth Wilson, P.F. | 1 | 0 |
| Annie Lewis, P.F. ... | 1 | 0 |
| E. Bellamy, P F. ... | 1 | 0 |
| M. M. Curtis, P.F. ... | 2 | 6 |
| C. E. Knights, P.F. ... | 1 | 0 |
| S. C. Townshend and Friends, P.F. ... | 2 | 0 |
| C. Charlton, P.F. ... | 5 | 0 |
| J. Lawrence, P.F. ... | 1 | 0 |
| Policy 3,550 ... ... | 5 | 0 |
| Agnes Burnett, P.F.... | 5 | 0 |
| Annie E. Andrews ... | 5 | 0 |
| F. Low, P.F. ... ... | 5 | 0 |
| B. Lancaster, P.F. ... | 5 | 0 |
| Emma Rouse, P.F. ... | 3 | 0 |
| F. A. Kenyon, P.F. ... | 2 | 6 |
| Helen B. Legg, P.F.... | 2 | 6 |
| Miss Jessie Gowan, P.F. | 2 | 6 |
| Alice Whiteside, P.F... | 2 | 6 |
| S. A. Clark, P.F. ... | 2 | 6 |
| J. Pye, P.F. ... ... | 2 | 6 |
| P. Waite, P.F... ... | 2 | 6 |
| E. A. Manley ... ... | 2 | 6 |
| L. Manley ... ... | 2 | 6 |
| G. Manley, P.F. ... | 2 | 6 |
| Agnes Duncan ... ... | 2 | 6 |
| Policy 1,988, P.F. ... | 2 | 6 |
| Mary Love, P.F. ... | 2 | 0 |
| E. Needley, P.F. ... | 2 | 0 |
| Policy 2,929, P.F. ... | 2 | 0 |
| Nurse Harley, P.F. ... | 2 | 0 |
| E. J. Rowe, P.F. ... | 2 | 0 |
| Amie E. Sing, P.F. ... | 1 | 0 |
| Policy 2,834, P.F. ... | 1 | 0 |
| Nurse Rush, P.F. ... | 1 | 0 |
| Policy 4,176, P.F. ... | 1 | 0 |
| F. Dell, P.F. ... ... | 1 | 0 |
| L. J. Mason, P.F. ... | 1 | 0 |
| E. A. Pollard Lewis, P.F. ... ... ... | 5 | 0 |
| Amy C. Lindsay, P.F. | 5 | 0 |

| | s. | d. |
|---|---|---|
| Ellen E. Hughes, P.F. | 5 | 0 |
| E. M. Warner, P.F. ... | 5 | 0 |
| K. E. Seabrook, P.F.... | 5 | 0 |
| Annie E. Mallett, P.F. | 3 | 0 |
| Nurse Cooper, P.F. ... | 3 | 0 |
| Julia Cox, P.F. ... | 2 | 6 |
| E. C. Slinger, P.F. ... | 2 | 6 |
| Nurse Ayre, P.F. ... | 2 | 6 |
| E. Cadwallader, P.F.... | 2 | 0 |
| A. L. Middleton, P.F. | 2 | 0 |
| E. Butler, P.F... ... | 2 | 0 |
| M. C Earp, P.F. ... | 2 | 0 |
| M. J. Gerrie, P.F. ... | 2 | 0 |
| Nurse James, P.F. ... | 2 | 0 |
| Martha Clegg, P.F. ... | 1 | 6 |
| Lucie Hill ... ... ... | 1 | 0 |
| S. A. Scott Palmer, P F. ... ... ... ... | 1 | 0 |
| Policy 7,302, P.F. ... | 1 | 0 |
| Policy 3,160, P.F. ... | 1 | 0 |
| Adeline Camm, P.F. ... | 1 | 0 |
| Miss Hobhouse ... ... | 5 | 0 |
| L. Ball, P.F. ... ... | 5 | 0 |
| H. Johnson, P.F. ... | 5 | 0 |
| Miss Kirkpatrick ... | 5 | 0 |
| M. L. Downes ... ... | 5 | 0 |
| Edith Day ... ... ... | 5 | 0 |
| Margaret Perceval, P.F. ... ... ... ... | 4 | 6 |
| Nurse Heygate ... ... | 4 | 0 |
| Julia Roberts, P.F. ... | 4 | 0 |
| F. M. Bates, P.F. ... | 4 | 0 |
| Emma E. Howleston, P.F. ... ... ... ... | 4 | 0 |
| M. L. Petrie, P.F. ... | 3 | 6 |
| Nurse Matthews, P.F. | 3 | 6 |
| F. E. Jones, P.F. ... | 3 | 0 |
| Annie Ling, P.F. ... | 3 | 0 |
| E. G. Sidley, P.F. ... | 2 | 6 |
| Nurse Foster ... ... | 2 | 6 |
| A. C. Brown, P.F. ... | 2 | 6 |
| Policy 289, P.F. ... ... | 2 | 6 |
| Nurse Wigley ... ... | 2 | 6 |
| M. E. Knight ... ... | 2 | 6 |
| S. Davidson, P.F. ... | 2 | 0 |
| M. Groom, P.F. ... ... | 2 | 0 |
| Nurse Roberts ... ... | 1 | 0 |
| Helen Booth... ... ... | 1 | 0 |
| E. S. Burnside Johnson | 5 | 0 |
| H. E. Smith ... ... | 5 | 0 |
| Lily Greenwood, P.F... | 5 | 0 |
| F. Hollis ... ... | 3 | 6 |
| H. Dickinson, P.F. ... | 3 | 0 |
| F. H. Frost ... ... | 2 | 6 |
| E. Allen, P.F.... ... | 2 | 6 |
| Lilias Robertson ... | 2 | 6 |
| Clara Cooper ... ... | 2 | 6 |
| Mary Haugh, P.F. ... | 2 | 6 |
| Policy 3,800, P.F. ... | 2 | 6 |
| Policy 4,550, P.F. ... | 2 | 0 |
| Policy 1,399, P.F. ... | 2 | 0 |
| Ellen L. Watts, P.F... | 2 | 0 |
| Policy 3,978, P.F. ... | 1 | 0 |
| Policy 4,360, P.F. ... | 1 | 0 |
| M. M. Blau ... ... | 1 | 0 |
| Janet A. Horsted ... | 1 | 0 |
| Emily Pratt, P.F. ... | 1 | 0 |
| Rosamond Candy, P. F. | 1 | 0 |
| Eleanor Lilly, P.F. ... | 1 | 0 |
| E. M. Parham, P.F. ... | 1 | 0 |
| A. M. Lane ... ... | 2 | 6 |
| Jane Walker ... ... | 2 | 0 |
| G. E. Jones ... ... | 1 | 0 |

Collected at the Royal Hospital, Putney.

| | s. | d. |
|---|---|---|
| Nurse Bell ... ... | 5 | 0 |
| Nurse Fidler ... ... | 2 | 0 |
| Nurse Aldred ... ... | 4 | 0 |
| Nurse Howard ... ... | 1 | 6 |
| Nurse Goulden ... ... | 1 | 6 |
| Nurse Smith ... ... | 1 | 0 |
| Nurse K. Butler ... | 1 | 0 |
| Nurse Grice ... ... | 1 | 0 |
| Nurse Parker ... ... | 1 | 0 |
| Nurse R. E. ... ... | 1 | 0 |
| Nurse M. S. ... ... | 1 | 0 |
| Nurse Acland ... ... | 1 | 0 |
| Nurse Roberts... ... | 1 | 0 |
| Nurse F. E. J.... ... | 1 | 0 |
| Nurse A. M. ... ... | 1 | 0 |

### Nursing the Wounded in the American Civil War.

Miss Louisa M. Alcott as a Nurse.

The author of "Little Women" and "Good Wives" is well known to all as one of the most charming and popular writers for young girls which the past half-century has produced, but the fact that she served as a military nurse for some time during the great American civil war is remembered by few. Soon after she had attained the height of her popularity she published a paper-bound volume relating her nursing experiences, which has been little read, but which, at the present juncture, is especially full of interest to the nursing world, giving, as it does, a vivid and somewhat startling picture of American hospital life and ways in the early sixties.

The Method of Enlistment.

This was delightfully simple. No papers to fill up, no interviews with matron or doctor, no uniform to procure, no vacancies to wait for. Miss Alcott wrote to a Washington hospital offering her services, and, enclosing a recommendation from a nurse whom she had known for two days (!), received her "commission" by return post, and set off at once.

Early Experiences.

For the first three days after her arrival at the hospital—which appears to have been a general institution, with some wards set apart for the wounded soldiers who were hourly expected—this brand new nurse was occupied by "the superintendence of a ward containing forty beds, where I spent my shining hours washing faces" (a good deal may be read between the lines here; and some light is cast upon subsequent remarks about smells), "serving rations, giving medicine, and sitting in a very hard chair, with pneumonia on one side, diphtheria on the other, five typhoids on the opposite, and a dozen dilapidated patriots hopping, lying, and lounging about." Comment on such a collection of cases would be superfluous, but one cannot help wondering how many of the "dilapidated patriots" escaped the all but universal "wound fever" of the day, which puzzled so many worthy surgeons.

How the Wounded Were Received.

One morning the wounded from Gettysburg arrived in a train of ambulances, and Miss Alcott was sent for to the ward where the new patients were being tumbled down in heaps over the floor as well as on the beds. "The first thing I met was a regiment of the vilest odours that ever assaulted the human nose . . . and the worst of this affliction was everyone had assured me it was a chronic weakness of all hospitals (!) and I must bear it. I did, armed with lavender water, with which I besprinkled myself and premises."

Matron's Instructions.

The matron then called up her three-day-old recruit, and quite appalled the latter by ordering her to "tell them to take off socks, coats, and shirts, scrub them well, put on clean shirts, and the attendants will finish them off and lay them in bed." Miss Alcott confesses to having been "staggered," but, "having resolved, when I came, to do everything I was bid, I drowned my scruples." Some of the patients, she avers, looked "grimly scandalised," but she finished her task with the aid of a "basin, sponge, and soap" (a bath being evidently not included in the paraphernalia of the hospital), and afterwards assisted to feed the patients, the menu, for some scores of dangerously wounded men, being hot bread, soup, meat, and coffee.

Miss Alcott's Ward.

The hospital was now apparently full to the brim, but Miss Alcott's ward—which held forty patients before the addition of the wounded—had no extra nurses granted to it. Miss Alcott, now on night duty, took entire charge all night, with some slight assistance from the watchman. A certain "black-eyed widow" took the day duty, and between them this totally untrained pair did an amount of work that would, as the authoress herself declared, be sufficient to break down a horse. The general watchman's duties appear to have been those of attending to "the fires and the wounds, the latter needing constant wetting."

Ventilation and Diet.

The windows of the wards were very rarely opened, for the upper sashes were nailed fast. The lower were considered too draughty to raise in winter weather. Miss Alcott contemplated the strong measure of breaking a few panes more than once, but never dared to carry out the deed.

The nurses' food consisted of the following, week in and week out: Salt beef, pork, salt butter, bad army bread, stewed blackberries, bad coffee, and tea made with "visibly animated" water. If a nurse in that happy-go-lucky establishment chanced to be so busy at any meal-time that she could not get away—rather a common occurrence—she went without that particular meal and starved till the next.

The Difficulties of Nursing.

Let the nurse of to-day, familiar with perfect ward routine and orderly arrangement of all things needful, pity her predecessor of the sixties, who had to go through such scenes as the following:—

"I am dressing Sam Dammer's shoulder, and having cleansed the wound, look about for some strips of adhesive plaster to hold on the little square of wet linen which is to cover the gunshot wound. The case is not in the tray. Frank, the sleepy, half-sick attendant (he was a convalescent with serious heart disease) knows nothing of it; we rummage high and low; Sam is tired and fumes; the men advise, and laugh at the flurry." An attempt to borrow some plaster from the next ward is met with a severe repulse. "I fly to the surgery. Mr. Toddypestle informs me that I cannot have anything without an order from the surgeon of my ward. Great heavens! where is he? . . . At last I find him, in a state of bliss over a complicated amputation, in the fourth storey. I make my demand; he answers, 'In five minutes,' and works away. . . . The five minutes grow to fifteen; Dr. P. tears himself away long enough to scribble the order." . . . Miss Alcott hurries to the surgery, finds it locked, and discovers the dispenser drunk in the dead-house. She worries the key from him, gets her plaster, and returns at last to the patient. "Just as I get him done, struggling the while with a burning desire to clap an adhesive strip across his mouth, full of heaven-defying oaths, Frank takes up his boot, and exclaims, 'I'm blest if here ain't that case now! I saw it pitch in this morning, but forgot all about it till my heel went smash into it. Here, ma'am, ketch hold.'" And out of the boot goes the plaster, straight to a gunshot wound!

Relatives Who Remained.

Another disagreeable side of hospital life in those days was the forcible invasion of the wards by relatives, who came determined to nurse their belongings, and stayed in spite of all opposition. In Miss Alcott's hospital this practice was not generally allowed, but a stray relative succeeded in running the blockade occasionally. One mother of a badly-wounded "case" slept on the floor of the ward, brought a daily basket "filled with luxuries for her boy" (most nurses know those luxuries!) to which no one apparently objected, and "pecked and cackled" at everyone who interfered, until the lad was ready to go.

Ignorance of Nurses.

It is hard to imagine a great hospital now-a-days served largely by nurses who had never seen an operation, and could not look on or assist at one; no one, apparently, expecting such a duty from anyone who did not feel called upon to volunteer. Such, however, seems to have been the case in Miss Alcott's institution; and that lady evidently earned no small glory from her willingness to look on when important surgical work was being done. Actual assistance was apparently never expected from, or given by, a nurse; operations generally being considered outside her department. "Several of my mates," Miss Alcott says coolly, "shrunk from such things, for though the spirit was wholly willing, the flesh was inconveniently weak."

### Miss Alcott's Salary.

The authoress's career ended, before many months, in a sharp attack of typhoid, after which she was invalided home. The salary she received, commencing with her entrance into hospital, amounted to £2 a month.

### Her Own Qualifications.

Reading between the lines one comes to the conclusion that Miss Alcott was a plucky, hard-working, enthusiastic nurse, rather averse to authority, and decidedly given to literary-sentimental views of her patients; not nearly strong enough for the life she undertook, and—like all women of her generation—counting it a virtue to deprive herself of necessary food and sleep at the risk of imminent breakdown. One fancies that the doctors may have found her a little trying at times through the much-deprecated *trop de zèle*. She seems to have had very definite views of her own regarding the treatment of her patients, and "more than once expressed a difference of opinion regarding sundry messes it was my painful duty to administer." However, in a day when nurses coming on trial "fainted at the sight of blood, were afraid to watch alone, couldn't possibly take care of delirious persons, were nervous about infections, and unable to bear much fatigue," Miss Alcott must have taken rank as a nurse of the very highest ability and value.

### Minor Appointments.

Isolation Hospital, Winchmore Hill.—Miss Jessie Pick has been appointed Night Sister. She was trained at the Hospital for Women, Soho Square, and has since been charge nurse at Brook Hospital, Shooter's Hill, charge nurse at Stockport Union Hospital, and sister at the New Infirmary, Birmingham.—Miss Annie Campbell has been appointed Day Sister. She was trained at the Royal Surrey County Hospital, Guildford, and has since been charge nurse at the South-Western Hospital, Stockwell, and district nurse at Leavesden Green.—Miss Enid Wheelwright has been appointed Staff Nurse. She was trained at Tiverton Infirmary, and has since held appointments as nurse at the Sanatorium, Wellington College, and staff nurse at Camelia Hospital, Poole.

Devon Infirmary, Barnstaple.—Miss Annie Ball has been appointed Charge Nurse. She was trained at the Royal Free Hospital, Gray's Inn Road, and has since been for three years charge nurse at the North-Eastern Hospital, Tottenham, and for sixteen months charge nurse at the Park Hospital, Hither Green.

Warrington Infirmary.—Miss Kathleen Bowyer has been appointed Staff Nurse. She was trained at the Royal Infirmary, Manchester. Miss Alice Holroyd has been appointed Staff Nurse. She was trained at Ancoats Hospital, Manchester.

Blaby and Wigston Magna Infectious Hospital, Leicester.—Miss Lucy Drew has been appointed Staff Nurse. She was trained at the Monsall Hospital, Manchester, and has since had charge of the Isolation Wards, Sunderland, and of the Isolation Hospital, Hinckley.

Durham County Hospital.—Miss K. Marie Johnsen has been appointed Sister. She was trained at the Royal United Hospital, Bath, and has since been night sister in the same institution.

Isolation Hospital, Muswell Hill.—Miss Ethel M. Stronach has been appointed Staff Nurse. She was trained at Plaistow Fever Hospital, West Ham.

### Nursing in West Africa.

### MISS MARY KINGSLEY ON THE COLONIAL NURSING ASSOCIATION.

In the course of a lecture at the Livingstone Exhibition on "The Betterment of the White Man's Life in West Africa," Miss Mary Kingsley said that, as there was not one of the conditions which could not be improved, the subject was so great that she could not possibly deal with all its branches. She would therefore urge the claims of the Colonial Nursing Association. This was part of Mr. Chamberlain's noble scheme to stop the waste of life that went on in the Tropics, and both for Imperial and sympathetic reasons it should be supported. Only those who had seen for themselves could realise of what benefit a scientific doctor and good nurses could be in a region like West Africa. What she thought was wanted there was a hospital cruiser with a staff of women nurses and two doctors. In connection branch hospitals should be established in the shore settlements to deal with those white patients who could not immediately be got on board, though they should always be sent there as soon as possible. The difficulties of hospital ships were known to her. Although she admitted that bilge was bad, she did not think it was fatal if properly managed. Again, there was the objection of the vessel being moored at sea. But the West African sea was not rough as a rule, except for tornadoes. When these came they relieved the monotony of life in a hospital ship, and it was possible to get used to never finding a thing where it had been left, owing to its displacement by the roll of the vessel lying broadside on to the oily swell. All the disadvantages of hospital ships together would not, she believed, equal the balance of advantages they would afford in the way of healthiness, and even the hospital hulk she thought was superior to coast sanatoria. As an alternative she suggested that the mail steamers trading with West Africa should be fitted with a roomy hospital cabin and provided with trained nurses, to act in concert with the shore hospitals.

### The Present Conditions.

She proceeded to discuss the problem of establishing an efficient nursing service under present conditions. Lady nurses could only touch the fringe of the work, owing to the difficulties of transport and the sparse and scattered population, which was a drawback to their efficiency. The Empire could not do much for the men in the hinterland bush, who were doing good work in its behalf, but they might be helped by good cooking and an acquaintance with the rules of hygiene. A doctor in a settlement told the people where to build their houses, &c., and generally had a prophylactic effect, which she hoped would also be exerted by trained nurses. It seemed a reproach to us at home that many a man's life was wrecked, and many a death caused, by the want of a little nursing.

### Practical Suggestions.

In West Africa both trained men and women nurses were required—the men for the up-country stations and the women in the shore ones—and she suggested that soldiers who had had experience of hospital duty in the South African campaign should be allowed the chance of acting in such a capacity at good salaries. There should be a good hospital staff at Sierra Leone and a place of call for ships in the Bight of Benin so that invalids might be cared for and made strong enough to face the trade winds. No very large army of nurses was wanted, but the staff should be a sufficient one, and a double one—to enable one set to be at home while the other was on duty, with a few extra ones to spare. A greater amount of leave was necessary for the nurses than for Government officials and those engaged in commerce, because their life in hospital was more dangerous to health. In advocating men hospital orderlies she was not under-rating the work of lady nurses. But the usefulness of the latter depended on what kind of women they were, and some who were very good here might not be so there, and *vice versâ*. Hence the advantage of a body like the Colonial Nursing Association to make the selection.

### Echoes from the Outside World.

### AN OPEN LETTER TO A HOSPITAL NURSE.

Monday was a very anxious day. The morning's papers contained the heliograph messages received from General White by General Buller announcing that he was hard pressed, that the Boers had been assailing him for hours in large numbers, and, reading between the lines, we naturally anticipated the worst. Then all day rumours kept flying about persistently that Ladysmith had fallen, and we dared not believe a report circulating in the Frere Camp that the defenders in Ladysmith had later in the day successfully beaten off the attack. The sun had failed for the time, and no more messages could be got through. But towards evening a blessed relief came to the almost intolerable strain which had lasted all day. The report for once had been a true one. Ladysmith was still held by the English, though the victory must have cost the poor garrison dearly. An entrenchment on Wagon Hill was three times taken by the enemy, and three times retaken by us at the point of the bayonet. Our casualties are as yet unknown, though the Earl of Ava is mentioned amongst those dangerously injured. A serious aspect of the affair is that a prolonged engagement means a much reduced supply of ammunition, a greater number of wounded to look after, Boer prisoners to feed, and no greater chance than before of the arrival of a relieving force. If Sir Redvers Buller could not even take advantage of the withdrawal from Colenso of large numbers of the enemy's troops to assist in the assault at Ladysmith, it obviously means that he is very much hampered. He simply shelled the position, but made no attempt to cross the river, or in any way to score a success. Probably he had excellent reasons for sitting still, but one trembles lest all the bravery should be in vain, and the poor little town fall at last from want of food and ammunition.

Private letters from South Africa, written, of course, three weeks ago, but only received this mail, tend yet further to sadden one, because the writers speak in such confident strains of the great things which Sir Redvers Buller was to do; how the preparations for a big battle were being carried forward, with 1,200 Rand refugees engaged as stretcher-bearers, 1,000 extra beds prepared, and ships made ready to receive the prisoners. Evidently, many of the colonials thought, as the Boers feared, that General Buller's arrival and action would prove of the utmost immediate importance. But, as we know, luck was against him, and the consternation which his repulse created here must have come with almost overwhelming force to the poor Natalians. The letters also speak most hopefully of the prospects of the war coming to a close, for though the writers confess that they had no heart for Christmas or New Year's festivities, they look forward to Eastertide, when they believe that their volunteer sons and brothers will have returned to them. But as the war progresses the end seems to grow farther and farther off, or so it seems to us in England. The anxiety of people in the larger towns in South Africa is naturally very great, because owing to the severe Press censorship it is so difficult to obtain news, but their position is enviable when compared with that of the Boer women. There appears to be no regular system at all for furnishing information to the Boer wives and mothers, and my correspondent says that they are nearly mad with anxiety. They clamour for news, but can get none, except accounts of victories, with no particulars of the names of killed and wounded. This statement, curiously enough, comes through a nurse, though not an English one. A little child in Durban, seriously ill with throat trouble, required trained nursing. The only nurse who could be found was a Cape woman married to a Hollander. Her husband was at Johannesburg, and her sympathies were all with the Boers.

The English Education Exhibition at the Imperial Institute, which will be open till the 27th, is hardly likely to be extensively visited by outsiders, yet there is much which should be intensely interesting, not only to those whose lot it is to teach the young idea to shoot, but also to all who are anxious to keep pace with the times and to know something of modern methods and modern achievements. Unquestionably, our grandmothers, if they could return to earth for a spell, would open their eyes exceedingly wide to find the specimens of bead work, brush work, and paper-folding which are shown as the results of infantile school labour, though they might regard the training of the hands with more favour when they passed on to note the cleverness of the exhibits in wood carving, inlaying, and metal work, due to the systematic development of the manual, as well as the intellectual, faculties. The London School Board arrangements for teaching domestic economy, including the making of beef tea and barley water for the sick, the cleansing and starching of the family wash, and a certain knowledge of sanitation, would probably find sufficient favour in their eyes. It is a trifling consolation, at all events, to the ratepayers to see some of the fruits of the heavy burden imposed upon them in the excellent work done by the children of the metropolis.

One point comes out very strongly in Mr. Balfour's speech at Manchester on Monday, namely, that the reason why the Government did not make preparations for the war early in the summer was not because they had much hope of peace being permanently established, but because the general drift of opinion would have been against them, and they would have been blamed if they had assumed a more warlike attitude. But surely there are times when the Government should at all costs take the course that seems best to them in the interests of the country. If they had fallen in the discharge of duty they might still have found that the path of duty was the way to glory. Mr. Balfour very properly protested—yet in vain—against the criticism of Generals in the field; and he made it very clear that the Cabinet do not intend, whatever happens, to throw individual members to the wolves.

On Saturday, for the first time for some weeks, I went to the theatre. Somehow, South African events have been so absorbing that nothing in the way of amusement has seemed attractive; but it does no good to brood, and I quite enjoyed the delightful little piece at the Duke of York's Theatre. Apparently lots of other folks had also felt the need of cheering, for the house was crammed. "Miss Hobbs" is a capital play, written in Mr. Jerome K. Jerome's happiest vein—plenty of good fun, plenty of smart writing, and a very pretty little love story to boot. If all new women were as charming as Miss Evelyn Millard appears—even when she is in her most rabidly reforming mood—I think there would be plenty of men ready to fall in love with them rather than run them down. Decidedly, the fact that though Miss Hobbs is so very advanced in her ideas she continues to dress in a most womanly and tasteful manner is a great point in her favour. The hero, Woolf Kingsearl, played by Mr. Herbert Waring, is quite nice enough to make even his lectures pleasant hearing, because he looks so strong and capable that one feels involuntarily he could *do* as well as *talk* should the emergency arise; and Miss Susie Vaughan shines brilliantly as an aunt, who is a peacemaker as well as a matchmaker. One small incident considerably amused me. A married woman quarrels with her husband and leaves him. She vows never to return, and asserts that she shall at once become a nurse and assuage her grief by living for others. Her excitement ends by her flying into a passion and entirely losing her self-control, thus showing her fitness for a post which requires, above all things, an absence of hysterical emotion, and a cool and calm forgetfulness of self. A month's trial would probably have convinced the poor ill-used wife that some things were even worse to bear than husbands who put bloomer costumes on the kitchen fire because they were so unbecoming to the woman of whom he was genuinely proud.

### The Prevention of Bed-Sores.

### EXAMINATION QUESTIONS FOR NURSES.—RESULT OF THE DECEMBER COMPETITION.

### The First Prize.

"South London" is the winner. Both she and the winner of the second prize are very near together in the merits of their answers. "South London" comes out first because of her intelligent grasp of the cause of bed-sores, a knowledge, I am sorry to say, by no means invariably displayed in the three hundred and odd other papers. She truly says that the friction itself is of far more consequence than the vehicle rubbed in.

### The Second Prize.

"Nurse Mary" wins this. She is not so emphatic on the necessity of restoring impeded circulation, but I gather from her answer that she understands that all other proceedings are useless without it, and she speaks of the advantageous effects of rubbing on—after washing and drying—a lather of soap. Many candidates speak of this plan, but their ideas are entirely erroneous. They say that in washing it is well to rub in the lather of soap and not wash it off. Their intention may be correct, but according to their expressions it would appear that the same lather used for cleansing purposes, with the addition of dirt and perspiration, is to be steadily rubbed into the patient's skin. "Nurse Mary" objects to the use of powder, and I think it may be admitted that on the whole it is better to banish it; by closing the pores it has an evil effect, and when mixed with perspiration is apt to form cakes and exert a very deleterious influence.

### The Best Answer Sent In.

"Nurse Isabel's" answer is far before all others in excellence, and would naturally have gained the first prize; but, unfortunately, her paper is too long—a sad pity, for it could easily have been brought within the official limits. It will be well for all candidates to read carefully what she says and note all the points on which she dwells. As to the different applications used, nurses always have their own favourites, and "Nurse Isabel," like "South London," very wisely remarks that the rubbing is of far more importance than the vehicle rubbed in.

"In considering the best means for the prevention of bed-sores the nurse must first bear in mind the two primary factors of their causation, pressure and friction—the first producing a congested state of the circulation, with corresponding decrease of vitality in the tissues and skin; and the second, irritability and abrasion. Her attention, therefore, must be unremittingly directed to the prevention of these first conditions, remembering at the same time that certain classes of patients are more liable to bed-sores than others, notably the aged, the helpless, those who are emaciated, and those whose weight is great. The parts most likely to be affected are naturally those on which the weight of the body falls more prominently, *i.e.*, the lower part of the back, the hips, shoulder blades, elbows, and heels, while knees and ears must not be neglected. For the maintenance of the circulation in those parts gentle massage is of the first utility, if the patient is able to bear it. The skin should first be carefully washed with warm water and soap (the soft palm of the hand, not a flannel, being used) and dried with great care and gentleness. For purposes of stimulating and hardening the skin the nurse may then take a mixture of whipped white of egg and brandy (or whisky) in equal parts, and gently rub it into the skin, continuing her rubbing softly for three or four minutes after the latter is perfectly dry. It should then be dusted over with starch and boracic powder in equal parts, but the nurse must always bear in mind that her careful massage is of quite as much importance as the application she employs. As long as the skin remains healthy this treatment need only be applied night and morning, but if a tendency to redness still remains at the conclusion of the process it had better be repeated (omitting the soap and water) either at midday or when the necessary movement of the patient gives the nurse an opportunity. If the case is likely to be a long one, or from its nature or the delicacy of the patient's skin specially liable to bed-sore, the nurse must take early precautions to place her patient on a water bed, and by means of air cushions and pads, which she will make herself of varying shape and size, maintain watchful guard against all undue pressure. The patient s position in bed should also be varied as often as opportunity offers, the nurse remembering that to relieve the pressure on any given part a very slight readjustment sometimes suffices, and that it is by no means necessary to weary the patient every time by a complete change of posture. The pads mentioned above may be best made of alternate layers of cotton-wool and tow, finely drawn out, and covered with soft linen, a hole being left in the centre as in round air cushions. A variety of small pillows, filled in the same way, may also be made, and are very useful in relieving both pressure and weariness, while the wrapping of tender parts in cotton-wool is another extension of treatment. Obvious causes of friction (connected occasionally with some peculiarity the case offers) must be provided against. The draw sheet, lower sheet, and blanket must all be kept tightly drawn, absolutely free from rucks or crumbs, and fresh cool areas for the patient to lie on may be secured by moving the draw sheet from one side to the other as occasion offers. Absolute dryness, and the prompt removal of anything which becomes soiled (followed by careful cleansing and powdering of parts adjacent) are essentials."

### "South London's" Answer.

"A bed-sore means the death of a part. This condition is brought about by constant pressure, combined with a low state of vitality. The pressure interferes with the supply of blood that nourishes the part, hence it sloughs or dies The means of prevention are absolute cleanliness, particular attention being paid to projecting parts, such as the bottom of the back, shoulders, heels, and hips, if the patient has to lie on either side. After washing, friction should be used to stimulate circulation, methylated spirit, boracic and starch powder, or some lubricant, but the rubbing is far more important than what is rubbed in. Keep bed perfectly dry, and free from wrinkles by tucking sheets tightly in, after drawing quite smooth; if a draw sheet is used pin the corners with strong safety-pins, also the mackintosh. Be sure all crumbs are carefully brushed out of bed. Never let patient stay too long in one position if it is permissible to change it. Put patient on air or water cushion if possible; if these are not available ring pads may be made stuffed with tow or bran."

### Nurse Mary's Answer.

"Careful washing of the parts likely to be affected. After the skin is thoroughly cleansed and dried, make lather of soap and gradually work it in with the hand till completely absorbed; no drying with towel should be necessary. Then apply spirit (brandy or methylated), which should also be thoroughly rubbed in, not merely dabbed on. The rubbing should be continued gently till any tendency to redness has disappeared. This treatment seems to invigorate the skin, and the gentle and continuous rubbing is very soothing to the patient. The position should be frequently changed, and spirit applied whenever this is done. If change of position is not practicable the parts that are receiving pressure should be rubbed at least every two hours. The lather of soap is advisable four times in the twenty-four hours From experience I should advise avoidance of ointment and powder. They both tend to fill up the pores of the skin, and the latter to conceal any inclination to redness; in fact, imparting a fair and deceitful appearance. Careful attention to the bedclothes, avoiding rucks and crumbs, is also of the greatest importance."

### Other Answers.

Amongst the very large numbers sent in—in nine months the number of competitors has doubled—though on the whole good, the same fault is observable—want of thought and thorough grasp of the subject. Almost all begin by declaring that absolute cleanliness is the first thing to be observed in the prevention of bed-sores. Far be it from us to underrate cleanliness, but it is *not* the first necessity. The restoration of impeded circulation is the first and most important thing to be attended to. Impeded circulation causing low vitality in the parts, aided by other disadvantageous circumstances, causes bed-sores. Hence circulation must be restored. No doubt a large proportion of our candidates understand this perfectly, but have not expressed themselves well.

A second point to be noticed is their hurried desire to place all their invalids on water beds. That is all very well, but, alas! such luxuries are not always or, indeed, often to be had. The question was meant to refer to those whose circumstances precluded them from having anything but the ordinary bed and appliances. Again, if patients can be moved continually from one position to another there would seldom be any excuse for bed-sores. This question will be repeated later in the year, with the hope that more intelligence will be brought to bear upon it. A very large proportion of nurses speak of collodion being painted on the threatened spot. I thought this was an antiquated remedy long since exploded. Collodion has a tendency to wrinkle at the edges, but, what is far more serious, it precludes the use of the necessary friction, and hides effectually from sight the beginning of the danger spot.

Question for January.

Explain your treatment of bed-sores in their different stages. N.B.—You are not asked how to prevent them.

Rules.

The competition is open to all. Answers must not exceed 500 words, and be written on one side of the paper only. The pseudonym, as well as the proper name and address, must be written on the *same* paper and *not on a separate sheet.* Papers may be sent in for fifteen days only from the day of the publication of the question. Failure to comply with these rules will disqualify the candidate for competition. Prizes will be awarded for the two best answers. Papers to be sent to "The Editor," with "Examination" written in the left-hand corner of the envelope.

N.B.—The decision of the examiners is final, and no correspondence on the subject can be entertained.

### Everybody's Opinion.

[Correspondence on all subjects is invited, but we cannot in any way be responsible for the opinions expressed by our correspondents. No communication can be entertained if the name and address of the correspondent is not given, as a guarantee of good faith but not necessarily for publication, or unless one side of the paper only is written on.]

### OUR CLOTHING DISTRIBUTION.

The Matron of Paddington Green Children's Hospital, writes: I beg to acknowledge receipt of parcel of flannel and knitted vests sent from the "Clothing Distribution" for our convalescents, and thank you very much for so kindly thinking of us. The gift is much appreciated.

### THE NURSING OF TYPHOID.

"Matron" writes: In regard to the discussion in your paper about the disinfection of typhoid stools may I say that typhoid germs grow and thrive in carbolic lotion, and that in this specific fever perchloride of mercury is a good germicide to use (1 in 1,000). I believe also I am correct in stating that within a period of twelve hours typhoid excreta is not infectious provided it is in a liquid state. The smallest dry stain of a motion on linen or clothing is at any time infectious. This is a fact often lost sight of by good nurses, who are otherwise careful about disinfection in typhoid.

### NURSES' READING SOCIETY.

Miss Moberly writes: I am very pleased to be able to say that this society is beginning its third quarter with nearly seventy members. I was very sorry that so few nurses answered the papers upon our first quarter's book, "Tuberculosis," and hope that more answers may be forthcoming upon the books of the October quarter. Two books were set, to suit the convenience of the lending library, and half the members had "Mental Nursing," by W. Harding, whilst the other half had "Manual of Hygiene," by J. Glaister. The January quarter will see these books reversed, and the members who read the former volume last quarter will be reading the latter during the current quarter.

### A SPECIAL CORPS OF TRAINED NURSES TO VOLUNTEER FOR THE WAR.

Miss Lucy A. Smith, M.R.B.N.A., Felsham, Davenport Road, Coventry, writes: If a special corps of trained nurses should be organised to go to South Africa to nurse cases of enteric I should like to offer my services. I was for six years matron of Rochdale Fever Hospital, where one of our blocks (consisting of two large wards and two small ones) was set apart for the nursing of enteric patients, so that I have had a great deal of experience in the nursing of typhoid, and I know that the Medical Officer of Health (Dr. Henry) and all my Rochdale friends would gladly speak on my behalf.

"G. E. M." writes: I have read with pleasure "Sympathiser's" letter which appeared in last week's issue of The Hospital. I have had considerable experience in the nursing of enteric, and am a nurse of eight years' standing. If a special corps of trained nurses should be organised for South Africa I shall be most anxious to join it; but I am not in a position to pay my passage out or to give my services gratis. One reads of so many cases among our soldiers of enteric, dysentery, &c., and one wonders how the 56 nurses now engaged can possibly cope with the numbers now laid up, either from illness or wounds. I hope if any scheme is definitely settled that it will be duly published in The Hospital, and that all nurses who are able will respond heartily, and render the undertaking a success.

"An Officer's Daughter," M.R.B.N.A., writes: "Sympathiser's" letter expresses the thoughts of the majority. Can those suffering from typhoid, pneumonia, &c., be properly cared for under existing circumstances? If not, why should a special corps of nurses, volunteering to nurse such cases free of expense, not be selected? Men of all classes are volunteering to help their countrymen and country, leaving self-interest out of the question, and there are many women, who have small means, sufficient to live on in times of peace, and are thoroughly well trained nurses, who would only too gladly give their services for their country under authorised leadership. Expense is generally the barrier to all fresh schemes. Many trained nurses who have never taken up surgical work since training, or who have done other kinds of nursing, would make most trustworthy and hard-working medical nurses, and leave the army nursing more free to attend to those wounded, of whom we have only too much reason to fear there will be an increased number shortly. Could not someone like the Duchess of Portland, with whom we all sympathise, be induced to take up the subject? Let the nurses pass qualified committee of selection, and if it is not thought advisable to provide the necessary accommodation, let the nurses be placed at the disposal of the Government. Many of us have already worked in Government or British Consular service abroad, and would, I feel sure, prove as efficient as the Army Reserve men if called out. Scutari cemetery is full, not of those who died of wounds, but of disease and the effects of exposure. Can we not improve upon that in 1900?

### ST. GEORGE'S INFIRMARY, FULHAM ROAD.

"An Indignant Probationer" writes: In reference to the statement made in The Hospital on January 6th respecting a grossly mismanaged London infirmary, viz., St. George's Infirmary, Fulham Road, might I make a protest against a would-be friend. I have been a probationer here for over two years, and have never found patients or nurses' food inadequate, but plentiful and well served. Our matron and doctor take the greatest interest in their staff and endeavour to maintain the highest standard of nursing possible for their patients' comfort, and we have as a body the greatest confidence in the efficiency of our training school. Our grievance is overwork, and does not concern the public. In spite of our timidity (?) surely we might be allowed to plead our own cause. We have lodged our complaint in the right quarter, and feel sure of a redress shortly. Respecting damp linen I did not think we had one single nurse so devoid of good sense as to put on damp linen, and think I might venture to state if the Editor would come and see for himself he would find us fair specimens of underfed, timid, salicylic-taking probationers.

"A Third Year Probationer" writes: In reference to the article in last week's issue, headed "Gross Mismanagement at St. George's Infirmary" (rather a strong term, is it not?), I beg to thank the one who has so kindly interceded on our behalf, and to point out to you a few matters which are somewhat exaggerated. The nurses' food is good, varied, and well cooked, and from the patients there are no complaints. As regards the damp clothes, I believe once they were returned to the building (but not to the nurses) in that condition, but this received most prompt attention, and has not occurred again. Is it any wonder that out of 60 nurses a few have minor ailments at this time of year, and especially if they are subject to rheumatism, &c. I must say those nurses who are too nervous to go to the matron have my deepest sympathy. True, we have appealed to the committee, but not to the public.

"One of the Nurses" writes: I have been a probationer here one year and six months, during which time I have never had a complaint of the insufficiency of food from patients. As regards the nurses' food it is ample, good, varied, and well served. Referring to the nurses' laundry I think the statement is greatly exaggerated concerning the return of wet clothes. I am happy to say I can mention that the majority of the staff do not suffer from "rheumatism," and also, if necessity arises, I do not hesitate to say that we should not fear to consult the matron, as timidity does not reign among us. The petition presented to the committee from the nurses is entirely private, and surely they should be allowed to settle their own affairs without interference from outsiders. The matron is an excellent woman, and does all that lies in her power to make us happy and comfortable.

"Another of the Nurses" writes: I have been a nurse here nearly three years, during which time I have never had a complaint of the insufficiency of food from patients. The nurses' meals are ample, and the diet varied, good, and well served. Having been in two institutions of good reputation previously, I can speak with confidence. Referring to nurses' laundry, I think the statement rather exaggerated concerning the return of wet clothes, and am happy to say I can mention that the greater part of the staff do not suffer from "rheumatism," and also, if necessity arises, I do not hesitate to say that we should not fear to consult the matron. The timid ones (very few in number) have my deepest sympathy. The petition presented to the committee from the nurses is entirely private, and surely they should be allowed to settle their own affairs without interference from outsiders.

"Major" writes: Allow me to make a statement in opposition to that which appeared in The Hospital of last week in connection with the alleged mismanagement of St. George's Infirmary. Being at present a probationer in this institution, and having had previous experience in similar institutions, I have not met with such admirable discipline throughout as we have here. The head officials are aware of the fact that there is a deficiency of staff, and for some time they have been doing all in their power to remedy this. Nothing whatever concerning patients or nurses is allowed to be overlooked, everything having the strictest supervision of the matron and medical superintendent. Your correspondent has given the public the preposterous idea that the patients are grossly neglected. That is not so; they are constantly having attention and comfort available. They have only to ask for anything within reason and they have it. As regards the patients' food it is plentiful and good, and they have the option of changing it weekly. The nurses also have not the least occasion to complain of their food, it being variable, nutritious, in abundance, and served up well. The lack of "off duty" hours certainly does demand a little consideration. The officials cannot be expected to "reform" the establishment in as short a time as it takes to form "outside opinions." Would your correspondent like his or her family skeleton brought forth before the eyes of the public to see how the household is managed and their private business carried on? I wish "Correspondent" to know that the infirmary will bear inspection from end to end any day, and could compete with any similar institution in London for cleanliness and well-established discipline. One more remark before I close, that, from whatever source "Correspondent" gained the information, it is not by any means conventional.

### AN EASY SWINDLE.

"Once Bit" writes: Referring to the letter in issue of 16th ult., which unfortunately I only saw afterwards, on Thursday I received a visit from apparently the same woman, who described herself as a matron of a home for incurables at the West-end—a building I had often passed—in urgent need of a nurse. After saying she had been sent to me by a Deaconess of my acquaintance in another part of London, she engaged me to go to the institute on the following morning, ultimately borrowing £2 of me, because she had lost her bag in the train, and particularly wished to pay some accounts, as the committee would meet on Friday. On my arrival at the home I found no vacancy, and saw the real matron, who had received a visit from the same woman. When she had been over the building she promised a donation of £20 towards the funds, afterwards borrowing 10s., with the same excuse as in my case. She was a well-dressed woman, between 35 and 40, about 5 ft. 3 in., rather stout, pale, with a somewhat Chinese cast of countenance, but not bad looking, with dark eyes, and a quick business-like way of speaking. Perhaps this description may put others on their guard.

### LONDON AND PROVINCIAL HOSPITALS.

"Provincially Trained" writes: I should like to have the opinions of more of your readers on London hospitals versus provincial ones. I was myself trained in a provincial one, and perhaps may be prejudiced in their favour. I also have worked in a smaller hospital, and in each of these I found the work varied and the training excellent. Our matron (in the hospital in which I trained) was a splendid woman, and she was most careful that every nurse who left the hospital as trained was fully trained in every detail of general nursing, medical and surgical. Each nurse in turn was theatre nurse for two months under the personal supervision of matron. Since then I have done private work, and have been astounded to find so-called fully-trained nurses holding a three-years' certificate from a London hospital ignorant of the most ordinary details of a nurse's work. Taking one case, a three years' nurse from one of the largest London hospitals was sent to a private case, but returned in an hour's time, as she was unable to catheterise her patient, a lady. She had never been taught how to do it. Another one declined to take an operation case as she had never seen an operation. Each of these nurses held a three years' certificate. Invariably in selecting charge nurses or sisters a London trained nurse is chosen in preference to a provincially trained one. It seems slightly unfair.

### Presentations.

Poplar and Stepney Sick Asylum.—On Saturday, Miss S. E. Polden, late assistant matron, was presented with a handsome dressing-bag, silver-mounted fittings, from the nursing staff; a silver butter cooler from six members of her class; and a handsome trinket set from members of the domestic staff; the occasion being her retirement in order to fill the post of matron at the Royal United Hospital, Bath. Miss Polden will be much missed at the sick asylum, and the staff heartily wish her success and happiness in her new sphere.

### To Nurses.

**In order to increase and vary the interest in the *Mirror*, we invite contributions from any of our readers in the form of either an article, a paragraph, or information, and will pay a minimum of 5s. for each contribution. All rejected manuscripts are returned in due course, and all payments for manuscripts used are made at the beginning of each quarter, i.e., January 1st, April 1st, July 1st, and October 1st.**

### A Book and its Story.

### A DUBIOUS HERO.

"I BELIEVE in God. I believe also in Hell."

With these words begins the story of "The Years that Came After."* The person who utters them is Greta Tregarthen, a child whose untaught instincts convince her that the eternal goodness of God is incompatible with the existence of the endless tortures of hell, even though at the moment the story opens she is set up on a stool, a defiant, inglorious Galileo, to recant her inmost beliefs.

Though she did not know it till years after, Greta's father, Roger Tregarthen, a clergyman, had long ago come to the same conclusion, and given up the family living in consequence. But at the same time as this noble sacrifice to conscience he had made another sacrifice, which one may be excused for thinking quixotic and foolish. He had taken upon himself the responsibility of a cheque, to which his younger brother had forged their father's name. He had not been prosecuted; but he had been cast out by his family for this. One may be forgiven for refusing to admire this act as much as the author seems to do. In the opinion of all the characters of the story a doubt of the existence of a lurid hell seems to be regarded as being as heinous as any crime of which the law takes cognisance; but one may be excused for doubting if, at any time during the last twenty or thirty years—and one cannot put the date of the story farther back—a doubt as to eternal punishment, or even as to the divinity of Christ, has been reckoned by the world at large as an offence to be weighed in the same balance as forgery. And one may venture to doubt, also, if a man who is a husband and the father of two girl-children is justified in taking upon himself the guilt of a crime which must inevitably ostracise not only himself but those for whose welfare he has made himself responsible. Mrs. Reynolds evidently regards her heroine's father as heroic; but it is the heroism of a selfish man, who sets a conscious value on his emotions. As a rule, the burden of our own sins is as much as the majority of us can bear.

This point, however, does not affect the interest of the tale. Greta grows up bright, clever, rebellious; while Irene, her elder sister, is a model of all the ladylike virtues. The family have lived in a Welsh valley, isolated from all their former friends, and the girls grow up without that knowledge of life and the world which nowadays it is more difficult to avoid than to obtain. Lovers came, however, at least to Greta. First in the form of Ralph Owen, the eldest son of the neighbouring clergyman, at whose church the girls and their mother and governess worship—for Mr. Tregarthen does not affect church-going at all. But Ralph obtains a tutorship to a nobleman's son, and goes away for two years, leaving behind him a heart which he has moved for the first time to consciousness of itself. Roused now out of her girlish gaiety, Greta takes more interest in her peasant neighbours, and thereby makes acquaintance, first, with Nesta Lewis, a betrayed girl, and, secondly, with Anthony Weybridge, the new curate, who has "eyes like Ralph's." Anthony sees her for the first time when she is taking care of Nesta's baby in the absence of its mother, and promptly pictures her in his mind as a Madonna-like woman, the ideal pastor's wife, while she, "in love with love," is not unwilling to accept him as her future husband, notwithstanding the fact that she is always more in love with him when he is absent than when he is near.

But Nesta's story had caught Greta's imagination, and she wove it into a novel, called "God's Law and Man's." The subject of the story certainly was not new, but the treatment of it seems to have been fresh, and the novel was a great success, chiefly because it was marked by a fearless realism, the full import of which the authoress herself could not comprehend. The first blow to Greta's pride in her work comes when her father, who has hitherto allowed her full freedom in her reading, even permitting her a free study of the classics, whose frankness of language she has unconsciously echoed in her own work, forbids her to read "George Trent's" novel. "George Trent" is her pseudonym. Hard upon this comes her lover's declaration—made, of course, in ignorance of the authorship of the book—that "it ought never to have been written," and that "he would not like his wife to read it." When, at last, he is informed of the truth regarding the authorship of the book, he breaks off the engagement; but, unfortunately, he does more than that. When Weybridge asked for Greta's hand, Mr. Tregarthen had felt it his duty to tell his future son-in-law the secrets of his past life. Now, when the engagement between him and Greta is broken off, he has to write to the friend who was to have been best man at his wedding, and adds to the announcement the words, "I expect I was a fool to have anything to do with the family. The father, Roger Tregarthen, was himself, as I daresay you will have heard, the hero of a *cause célèbre* some twenty years ago." Anthony's friend must have been a journalist or an acquaintance of journalists, for soon after there appeared in a newspaper a long account of the identity of "George Trent," and also a recapitulation of her father's past misdeeds, real and reputed. The pain caused by this reopening of old sores may be imagined. Mrs. Tregarthen, a selfish, exacting woman, whose life had been a disappointment, makes Greta suffer for being the unconscious cause of this opening of the family skeleton's cupboard; while Irene takes a curiously mediæval view of matters for a girl who lives in the era of M.A.P.—we apologise, "P.A.M."—and whose sister's writings are supposed to resemble Hall Caine's. "Father will go to Hell—to Hell," she kept moaning; and then, with apparent inconsistency (should not this be "irrelevance"?), "I shall never marry now."

Yet Irene does marry; and her husband is no other than Anthony Weybridge, who gets over his disappointment and finds that the gentle and orthodox elder sister is much more suited to him than the brilliant but wilful Greta. In the end, too, Mr. Tregarthen becomes reconciled to his family, his father being convinced on his death-bed that his elder son had taken on his shoulders the burden of the younger's crime, so that he is rehabilitated both in fortune and reputation; and the story ends with Greta sitting by her father's side, assuring him that she will never leave him; a statement which, as it immediately follows Mr. Tregarthen's announcement that Ralph Owen, Greta's first lover, is in the neighbourhood, may be taken with some reservation.

Greta is a delightful heroine; not too much of a hoyden, nor yet of too modern and learned a cast, and the book as a whole is pleasant reading, though, as we have indicated, Mrs. Reynolds does not succeed in making us see her characters with her eyes. Mrs. Tregarthen, shallow creature as she was, had an undoubted grievance; not, indeed, in her husband's renouncing his position in the church when he felt he could no longer fill it with sincere conviction, but in his taking on his own shoulders the burden of a deed he was not guilty of, and the shame of which must react on his wife and children. And what can we think of the brother who let him be a scapegoat, accepting the sacrifice, not only in a moment of stress and terror, but living for ten years "the light of the old home," while his brother, who had taken the shame of his crime upon him, dwelt out in the wilderness? Mrs. Reynolds' philosophy tends to be morbid, and she forgets that though sacrifice may sometimes be the highest duty, it should be a sacrifice for the truth's sake, not for that of a lie.

* "In the Years That Came After." By Mrs. Fred Reynolds, Author of "Llanartro, a Welsh Idyll," "A Tangled Garden," &c. (Hutchinson and Co.)

### For Reading to the Sick.

"BLESSED is the man whom Thou chastenest, O Lord, and teachest him out of Thy law: that Thou mayest give him rest from the days of adversity, and that he may not be desolate in the earth."

Take my life, and let it be
Consecrated, Lord, to Thee;
Take my moments and my days,
Let them flow in ceaseless praise.

Take my hands and let them move
At the impulse of Thy love;
Take my feet and let them be
Swift and beautiful for Thee.

Take my voice and let me sing
Always, only for my King;
Take my lips and let them be
Filled with messages from Thee.

Take my silver and my gold,
Not a mite would I withhold;
Take my intellect and use
Every power as Thou shalt choose.

Take my will and make it Thine;
It shall be no longer mine;
Take my heart, it is Thine own,
It shall be Thy royal throne.

Take my love; my Lord, I pour
At Thy feet its treasure-store;
Take myself, and I will be
Ever, only, all for Thee!—*F. R. Havergal.*

**Reading.**

My son, I am the Lord Who gives thee strength in the day of trouble. Come to Me when it is not well with thee.

This is what most of all hinders heavenly consolation, that you are too slow in turning to prayer.

For before you earnestly seek Me, you seek oftentimes many other sources of consolation, and refresh yourself with external things.

And therefore it comes to pass that you get little assistance until you realise that I am He Who delivers those who trust in Me; and that without Me there is no real help, no profitable counsel, no lasting remedy.

If I send you affliction or any cross whatever, do not assume an injured tone, nor lose heart; because I am able quickly to raise you up again, and to turn all your heaviness into joy.

Where is your faith? Stand firmly and perseveringly, be patient and brave; comfort shall come to you in due season.

Wait for Me, wait; I will come and heal you.

It is a temptation which vexes you, and a vain fear which terrifies you.

What does your anxiety about future events—events which may never happen—bring you but sorrow upon sorrow?

"Sufficient unto the day is the evil thereof."

It is vain and profitless to entertain either joy or sorrow concerning future things, which perhaps may never come to pass.

If you are really wise, and if you take a true view of things, you ought never to give yourself up to despondency because of affliction, but rather to rejoice and give thanks.

Yes, account it a special reason for rejoicing that I afflict you with sorrows, and do not spare you.

"As the Father hath loved Me, I also love you," I said to My beloved disciples, whom indeed I sent not forth to temporal joys but to great conflicts; not to honours, but to contempt; not to ease, but to toil; not to rest, but to bring forth much fruit with patience.

My son, remember these words.—*Thos. à Kempis.*

### Notes and Queries.

**The contents of the Editor's Letter-box have now reached such unwieldy proportions that it has become necessary to establish a hard and fast rule regarding Answers to Correspondents. In future, all questions requiring replies will continue to be answered in this column without any fee. If an answer is required by letter, a fee of half-a-crown must be enclosed with the note containing the enquiry. We are always pleased to help our numerous correspondents to the fullest extent, and we can trust them to sympathise in the overwhelming amount of writing which makes the new rules a necessity.**

**Every communication must be accompanied by the writer's name and address, otherwise it will receive no attention.**

---

*Military Hospitals.*

(154) "A Reader," seeing the announcement in reference to Fort Pill (? Pitt) Hospital, Chatham, among the appointments in your issue of last week, would like to know if it is possible to be trained there, as she has been under the impression that Fort Pitt is purely a military hospital, where no training of probationers takes place.

No nurses are trained in military hospitals. Fully-trained nurses undergo a final course at Netley for the Army Nursing Service.

*Cookery Book.*

(155) Will you kindly tell me what is the best book on invalid cookery? —*W. H. A. T.*

There are cookery books by the score, and it is a matter of opinion which is the best. Messrs. Black have just published a second edition of the "Nurses' Handbook of Cookery" (price 1s. 6d.), by E. M. Wornsnap, which seems comprehensive.

*Lead Lotion.*

(156) I should be glad if you would enlighten me on the use of lead lotion. How does it act? Is it used as a cooling lotion or as a mild fomentation, and in what cases? I ask the latter question because I have been taught that a lotion is a cooling application, which should be applied on a single piece of lint and allowed to co-operate. I have, however, seen lead lotion in hospital applied with protection over it, and sometimes mixed with hot water. Why not in these cases have an ordinary fomentation? Can it be applied to a broken skin, and is it antiseptic?—*W. H. A. T.*

Lead lotion may be used as a mere evaporating and cooling lotion, in the same way as spirit lotion, like which also it is very mildly antiseptic. But lead lotion also has an action as a sedative and astringent, which operates equally when warm. As a soothing application, for example, the use of hot lead and opium lotion applied as a fomentation, and covered with oiled silk, is well known.

*Incurable.*

(157) Will you kindly tell me if there is any home where a paralysis case could be taken in after discharge from the National Hospital as incurable? The patient is a man about 60, quite paralysed, and almost blind. His friends are not well off, but might arrange to pay a small sum weekly.—*District Nurse.*

The Home for Incurables for the Border Counties, Strathclyde, Carlisle; the Yorkshire Home for Chronic and Incurable Disease, Harrogate; and the Alexandra Home for Chronic Invalids are all suitable.

*Air Cushions.*

(158) 1. Will any district nurse kindly say whether various sized air cushions can be inflated except by the mouth? 2. What is the most suitable way of disinfecting nozzles of cushions inflated by various mouths?—*Nurse Jane.*

The cushion could be easily inflated with a small pair of bellows, if the nozzle of the cushion and that of the bellows were joined by a bit of rubber tubing. 2. A thorough washing with soap, water, and a small brush ought to answer the purpose of cleansing the mouthpiece sufficiently; but if it be desirable to render it aseptic, it could easily be plunged in a carbolic solution of 1 in 20.

*Dispensing.*

(159) Would you kindly inform me if it would take me long to learn how to become a dispenser. I wish to get a situation as such in some institution. Any hint or information would be gladly received.—*Opia.*

A useful article appeared in THE HOSPITAL "Nursing Mirror" for July 1st, entitled "Dispensing up to Date," which gave a good idea of the work to be done. There was another in the "Nursing Mirror" for April 3rd, 1897, which went very fully into the best ways of qualifying oneself for the work. You should write to the Secretary of the Apothecaries' Hall, Blackfriars, for particulars of the assistant dispenser's certificate.

*Male Nurse.*

(160) Can you tell me where a male nurse may receive training in England?—*E. E. M.*

The National Hospital for the Paralysed and Epileptic, Queen's Square, Bloomsbury, is the only hospital that trains men for sick nursing. Mental attendants receive some instruction in the infirmary wards of asylums.

---

*Standard Books of Reference.*

"The Nursing Profession: How and Where to Train." 2s. net.

"The Nurses' Dictionary of Medical Terms." 2s. 6d. net.

"Burdett's Series of Nursing Text-Books." 1s. each.

"A Handbook for Nurses." (Illustrated.) 5s.

"Nursing: Its Theory and Practice." New Edition. 3s. 6d.

